# FKBP4 Accelerates Malignant Progression of Non-Small-Cell Lung Cancer by Activating the Akt/mTOR Signaling Pathway

**DOI:** 10.1155/2020/6021602

**Published:** 2020-12-04

**Authors:** Wen Meng, Jingfei Meng, Hong Jiang, Xing Feng, Dongshan Wei, Qingsong Ding

**Affiliations:** ^1^Department of cardiothoracic surgery, Affiliated Hangzhou First People's Hospital, Zhejiang University School of Medicine, Hangzhou, Zhejiang 310006, China; ^2^The Second Affiliated Hospital of Xi'an Jiaotong University, Xi'an, Shaanxi 710061, China

## Abstract

**Objective:**

To study the expression, biological function, and mechanism of FKBP4 in non-small-cell lung cancer (NSCLC).

**Methods:**

First of all, the expression of FKBP4 in NSCLC tissues and cell lines was detected by qRT-PCR; then, the effects of FKBP4 on proliferation, apoptosis, migration, and invasion of NSCLC were studied by CCK-8 assays, flow cytometry assays, wound-healing assays, and Transwell assays. After that, tumor xenografts were used to explore the effect of FKBP4 on NSCLC tumor growth in vivo, and the phosphorylation of Akt and mTOR was measured by western blot.

**Results:**

FKBP4 was highly expressed in NSCLC tissues and cells, and its expression was closely related to NSCLC tumor size, lymph node metastasis, and patient prognosis. In vitro, FKBP4 can promote NSCLC cell proliferation, migration, and invasion and inhibit NSCLC cell apoptosis. In vivo, FKBP4 can promote NSCLC tumor growth. Furthermore, FKBP4 can promote Akt and mTOR phosphorylation and activate the Akt/mTOR signaling pathway and an mTOR inhibitor can neutralize the functions of FKBP4 in NSCLC cells.

**Conclusion:**

FKBP4 serves as an oncogene to promote malignant processes in NSCLC, and it has the potential to be used as a biological marker and therapeutic target for NSCLC.

## 1. Introduction

Lung cancer is the most common cause of cancer-related deaths worldwide [1, 2]. Non-small-cell lung cancer (NSCLC) accounts for about 85% of lung cancer cases and is mainly divided into several common subtypes: squamous cell carcinoma, adenocarcinoma, and large cell carcinoma [3]. Although great progress has been made in chemotherapy and molecular targeted therapy for NSCLC, the 5-year survival rate of the disease is still lower than 15%, due to limited treatment options and tumor metastasis and recurrence. There is no doubt that a better understanding of the pathogenesis of NSCLC is essential to improve the diagnosis and prognosis of patients with non-small-cell lung cancer. In the occurrence and development of NSCLC, a variety of oncogenes and tumor suppressor genes are abnormally expressed, and they have been involved in the malignant biological process of tumor cells. Moreover, in recent years, many research and studies focused on oncogenes and tumor suppressor genes have been reported.

FKBP4, also known as FKBP52, is a member of the immunophilin family, which plays a role in immune regulation, protein folding, and transportation. The encoded protein of FKBP4 is a cis-trans-proline isomerase, which can interact with immunosuppressant FK506 and rapamycin [4]. Studies have shown that FKBP4 has a potential role in tumorigenesis and is considered as a possible biomarker. FKBP4 is expressed in most tissues, with the lowest expression in the breast, bladder, and testis [5, 6]. The expression of FKBP4 was elevated in several cell lines of hormone-dependent cancers, including breast cancer cell lines [7, 8] and prostate cancer cell lines [9]. Moreover, the expression of FKBP4 in breast cancer tissues and preinfiltration breast cancers was higher than that in normal breast tissues [8, 10]. Similar observations have also been made in prostate biopsy tissues [11] and liver cancer tissues [12], which indicates that FKBP4 might be a potential biomarker for tumors. Recent studies illustrated that the expression of FKBP4 is related to breast cancer progression and prognosis. It was illustrated that FKBP4 can promote the growth of triple-negative breast cancer cell models and xenograft tumor models [13]. It also reported that the amplification and overexpression of FKBP4 are potential mechanisms for castration resistance to prostate cancer development, and the interaction of FKBP4 with androgen receptors may provide potential therapeutic targets in prostate cancer [14]. Therefore, FKBP4 may act as an oncogene in tumors and promote the malignant progression of tumors.

In this study, first, it was found that FKBP4 expression was significantly upregulated in NSCLC through bioinformatics analysis, then verified by qRT-PCR in NSCLC tissues and cell lines, and finally, the clinical relevance of FKBP4 expression was analyzed. Subsequently, the function of FKBP4 in NSCLC cell lines and animals were investigated, and the relevant mechanisms of FKBP4 affecting tumor progression were also explored.

## 2. Material and Methods

### 2.1. Clinical Samples and Ethics

Primary NSCLC tissues and adjacent normal tissues were obtained from NSCLC patients who underwent surgical resection in our hospital. All patients did not receive radiation or chemotherapy before surgery. All tumor and adjacent normal lung tissue specimens were frozen immediately after resection and stored in liquid nitrogen until total RNA was extracted. All tumors and matched normal tissues were confirmed by pathology. The clinical characteristics of each patient were also collected, and written informed consent was obtained from all patients participating in the study. This study was approved by our ethics committee.

### 2.2. Bioinformatics Analysis

The Oncomine database (https://www.Oncomine.org/) was used to analyze the differential expression of the FKBP4 gene in large cell lung cancer, squamous cell lung cancer, and normal tissues. The Cancer Genome Atlas (TCGA) Lung Cancer (LUNG) database (http://cancer.genome.nih.gov/) was used to investigate the differential expression of the FKBP4 gene between lung adenocarcinoma and normal tissues. The significantly differentially expressed genes were determined if the fold change between tumor samples and normal samples is greater than 2 and *P* < 0.05.

### 2.3. Cell Culture

All NSCLC cell lines (A549, H1975, H358, and PC-9) and human bronchial epithelial cells (HBE) were purchased from ATCC. The cells were cultured in DMEM (Invitrogen); 10% fetal bovine serum (Invitrogen), 100 U/ml penicillin, and 100 mg/ml streptomycin were added. All cell lines were cultured in a humidified cell incubator with 5% carbon dioxide at 37°C.

### 2.4. Cell Transfection

The overexpressed plasmid and shRNA were transfected into the cells with Lipofectamine 3000 reagent (Invitrogen), and further experiment was performed after an appropriate period. FKBP4 shRNA was purchased from Santa Cruz Biotechnology; FKBP4 cDNA was amplified and inserted into the pCMV4 expression vector to obtain the overexpressed plasmid.

### 2.5. CCK8 Experiment

The CCK8 experiment was used to assess cell proliferation capacity. Cells were seeded in 96-well plates and cultured for 24 hours; plasmid or shRNA was transfected the next day. After 24, 48, and 72 hours of cell culture, the cell proliferation ability was measured using the CCK8 kit (GLPBIO, USA) and a spectrophotometer (450 nm).

### 2.6. Flow Cytometry Detection

Cells were seeded in 96-well plates and cultured for 24 hours; then, plasmid or shRNA was transfected the next day. 48 hours after the cells were transfected, the cells were collected, and cell apoptosis was detected using the Annexin V-FITC Apoptosis Kit (US BioVision) and flow cytometry.

### 2.7. Scratch Test

Scratch tests were used to assess the migration ability of cells. First, cells were seeded in 96-well plates and cultured for 24 hours; then, plasmid or shRNA was transfected the next day. 48 hours after the cells were transfected, a straight scratch was made on the monolayer of cells with the tip of a 10 *μ*l sterile pipette tip and washed three times with PBS. After the cells were cultured in serum-free DMEM for 24 hours, the migration of the cells was photographed using a microscope, and statistical comparison was performed.

### 2.8. Transwell Experiment

The Transwell assay was used to assess the invasion ability of cells. Cells were seeded in 96-well plates and cultured for 24 hours, and plasmid or shRNA was transfected the next day. 48 hours after cell transfection, cells were collected and counted. Invasion experiments were performed using a matrix gel-coated Transwell cell. First, 8 × 10^4^ cells were seeded with 100 *μ*l of serum-free DMEM in the upper chamber of the Transwell coated with matrix gel, and the lower chamber was filled with 500 *μ*l of complete medium containing 10% FBS. After that, the cells were cultured in an incubator for 24 hours. Finally, the cell was fixed with methanol and stained with crystal violet, and the number of cells passing through the membrane was counted.

### 2.9. Subcutaneous Tumor Formation in Nude Mice

The animal experiments in this study were approved by our ethics committee. BALB/c nude mice (male, 5-6 weeks, 18.0 ± 0.5 g) were obtained from Changzhou Cavins Experimental Animal Co., Ltd. First, the lentiviral-infected A549 cells (1 × 10^7^) with stably knocked out FKBP4 were injected subcutaneously in nude mice. Then, the tumor volume was measured every 5 days, and statistical analysis was performed. After the experiment, the mice were sacrificed by cervical dislocation.

### 2.10. Real-Time Quantitative PCR (qRT-PCR)

Total RNA was extracted from tissues and cells using Trizol reagent (Life Technologies), and 1.0 *μ*g of total RNA was reverse transcribed into cDNA with a total volume of 20 *μ*l using the PrimeScript RT Master Mix reverse transcription kit (Takara, Japan). Subsequently, PCR reactions were performed using the ABI 7900 system (Applied Biosystems, USA) with the SYBR Select Master Mix kit (Applied Biosystems) and 0.5 *μ*l cDNA. *β*-Actin was used as an internal reference, and the relative expression of FKBP4 was calculated using the 2^-*ΔΔ*Ct^ method. The primer sequences are FKBP4-F: GAAGGCGTGCTGAAGGTCAT, FKBP4-R: TGCCATCTAATAGCCAGCCAG Anti-pan-AKT antibody (ab8805, 1 : 1000, Abcam), anti-AKT (phospho T308) antibody (ab38449, 1 : 1000, Abcam), anti-AKT1 (phospho S473) antibody (ab81283, 1 : 5000, Abcam), anti-mTOR antibody (ab32028, 1 : 1000, Abcam), anti-mTOR (phospho S2448) antibody (ab109268, 1 : 1000, Abcam), anti-mTOR (phospho S2481) antibody (ab137133, 1 : 1000, Abcam), anti-S6K1 (phospho S424) antibody (ab131436, 1 : 1000, Abcam), anti-S6K1 antibody (ab32359, 1 : 1000, Abcam), anti-eIF4EBP1 (phospho T37) antibody (ab75767, 1 : 1000, Abcam), anti-eIF4EBP1 antibody (Y329) (ab32024, 1 : 1000, Abcam), and anti-beta-actin antibody-loading control (ab8226, 1 : 2000, Abcam).

### 2.11. Statistical Analysis

The results were analyzed using SPSS 19.0 software. Data was expressed as mean ± standard deviation. Statistical significance was compared between groups by the *t*-test and one-way analysis of variance. The chi-square test was used for clinical correlation analysis. All experiments were repeated at least three times; the difference is statistically significant when *P* < 0.05.

## 3. Results

### 3.1. FKBP4 Is Upregulated in NSCLC through Bioinformatics Analysis

**Figure 1:**
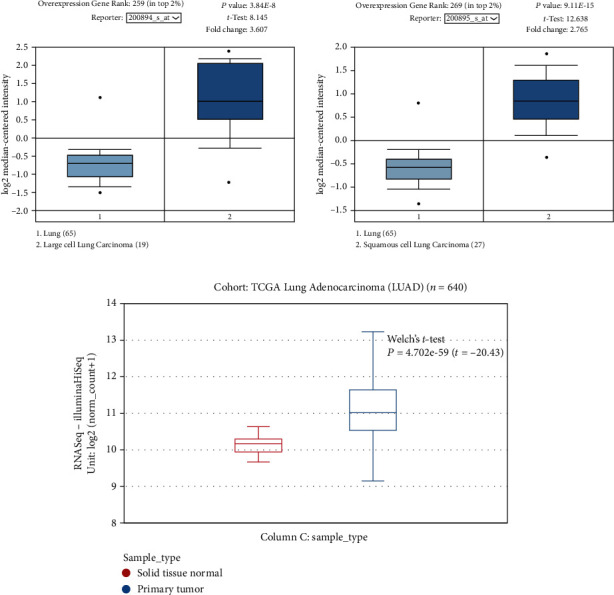
Bioinformatics analysis of FKBP4 expression in NSCLC: (a) expression analysis of FKBP4 in large cell lung cancer; (b) expression analysis of the FKBP4 gene in squamous cell lung cancer; (c) expression analysis of FKBP4 lung adenocarcinoma (*N* = 640, Welch's *t*-test, *P* = 4.702*e* − 59).

First, the Oncomine database was used to analyze the expression of FKBP4, and it was found that the expression of FKBP4 was upregulated both in large cell lung cancer (Figure 1(a)) and in squamous cell lung cancer (Figure 1(b)) compared with that in normal tissue. At the same time, the FKBP4 expression was also analyzed using TCGA Lung Cancer database and discovered that the expression of the FKBP4 gene was upregulated in lung adenocarcinoma (Figure 1(c)) compared to that in normal tissue. Through these bioinformatics analyses, we believe that FKBP4 is upregulated in NSCLC, and subsequent verification was performed.

### 3.2. FKBP4 Is Highly Expressed in NSCLC Tissues and Cells

**Figure 2:**
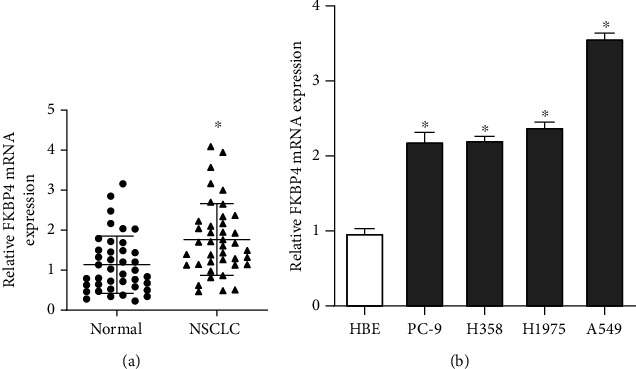
FKBP4 expression in NSCLC tissues and cells: (a) FKBP4 expression in NSCLC tissues, *N* = 40; (b) FKBP4 expression in NSCLC tissues; data were compared using ANOVA followed by the Dunnett *t*-test, *N* = 3; ^∗^*P* < 0.05 vs. normal or HBE.

40 pairs of NSCLC tissues and corresponding normal lung tissues were collected, and the expression of FKBP4 mRNA was detected by qRT-PCR. It was found that the expression of FKBP4 in tumor tissues was significantly higher than that in normal lung tissues (Figure 2(a)). Furthermore, it is discovered that the expression of FKBP4 was also higher in NSCLC cell lines than that in human normal bronchial epithelial cells (Figure 2(b)). These results indicate that FKBP4 is upregulated in NSCLC.

### 3.3. Clinical Significance of FKBP4 Expression

**Figure 3:**
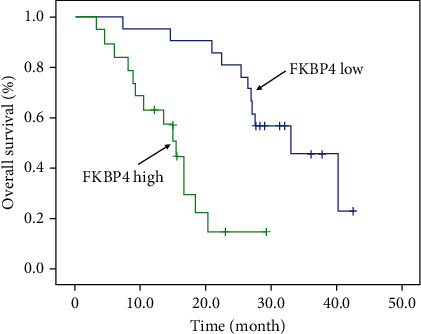
Survival analysis of patients with high and low FKBP4 expression groups; log-rank test, *N* = 40, *P* < 0.001.

Subsequently, the clinical data of 40 corresponding patients were collected, and the correlation between the clinical data and FKBP4 expression was analyzed. As shown in Table 1, FKBP4 expression is closely related to tumor size and lymph node metastasis. Moreover, through survival analysis, it was found that patients with high expression of FKBP4 had significantly shorter overall survival (Figure 3).

### 3.4. FKBP4 Overexpression and Silence Verification

**Figure 4:**
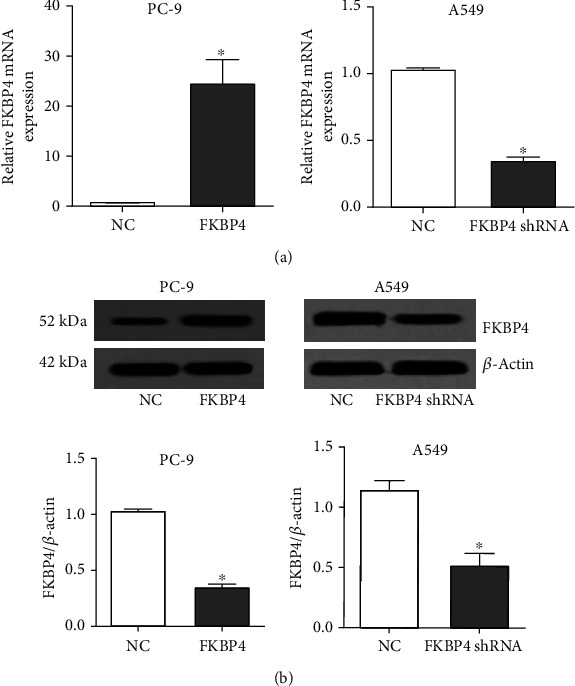
FKBP4 overexpression and silencing verification: (a) FKBP4 mRNA expression changes detected by qRT-PCR; (b) FKBP4 protein expression changes detected by western blot; *N* = 3, ^∗^*P* < 0.05.

Since among all NSCLC cell lines, the expression of FKBP4 in PC-9 cells is the lowest and the expression of FKBP4 is the highest in A549 cells, we transfected the FKBP4 expression plasmid in PC-9 and FKBP4 shRNA in A549 cells. qRT-PCR experiments and western blot experiments demonstrated the effectiveness of FKBP4 overexpression and silencing (Figures 4(a) and 4(b)).

### 3.5. FKBP4 Promotes Proliferation of NSCLC Cells

**Figure 5:**
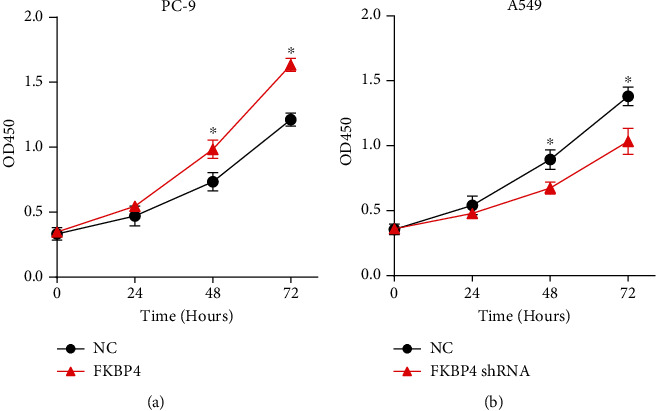
FKBP4 promotes the proliferation of NSCLC cells: cell proliferation after FKBP4 was overexpressed in PC-9 cells (a) or FKBP4 was silenced in A549 cells (b); *N* = 3, ^∗^*P* < 0.05.

After FKBP4 was overexpressed in PC-9 cells, it is found that the cell's proliferative capacity was significantly increased (Figure 5(a)) in the CCK8 experiment. However, after FKBP4 was silenced in A549 cells, the cell's proliferative capacity was significantly reduced (Figure 5(b)). These results indicate that FKBP4 can promote the proliferation of NSCLC cells.

### 3.6. FKBP4 Inhibits Apoptosis of NSCLC Cells

**Figure 6:**
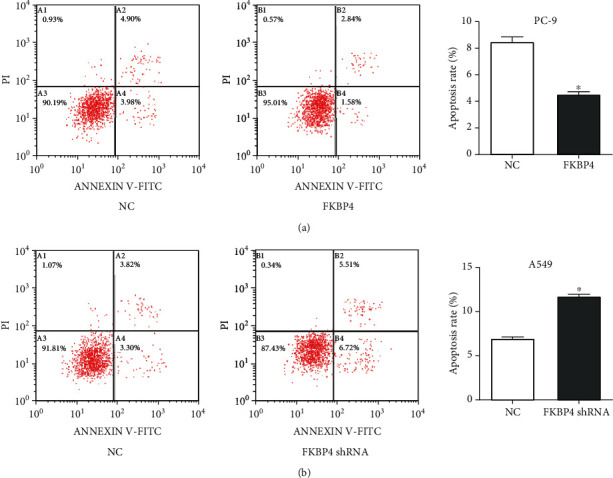
FKBP4 inhibits apoptosis of NSCLC cells: apoptosis of cells after overexpression of FKBP4 (a) in PC-9 cells and silencing of FKBP4 (b) in A549 cells; *N* = 3, ^∗^*P* < 0.05.

After FKBP4 was overexpressed in PC-9 cells, it is discovered that the apoptosis rate of the cells was significantly reduced in a flow cytometry experiment (Figure 6(a)), and after FKBP4 was silenced in A549 cells, the apoptosis rate was significantly increased (Figure 6(b)). These results indicate that FKBP4 can inhibit the apoptosis of NSCLC cells.

### 3.7. FKBP4 Promotes NSCLC Cell Migration

**Figure 7:**
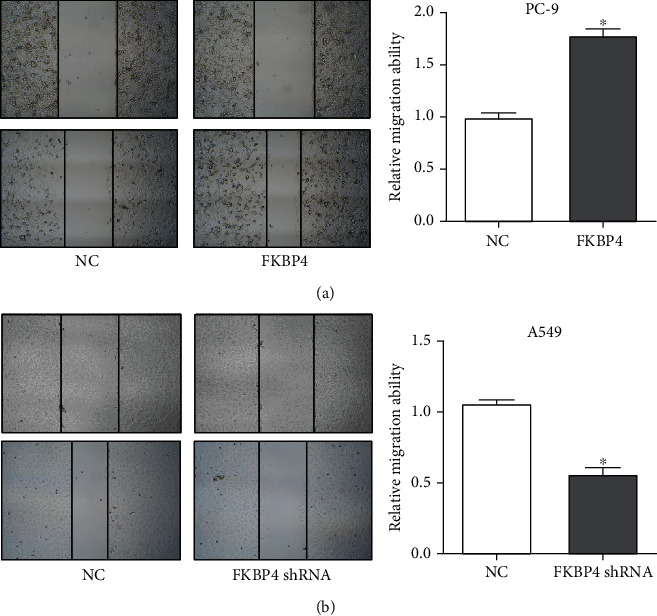
FKBP4 promotes NSCLC cell migration: cell migration after overexpression of FKBP4 (a) in PC-9 cells and silencing of FKBP4 (b) in A549 cells; *N* = 3, ^∗^*P* < 0.05.

After FKBP4 was overexpressed in PC-9 cells, it is shown that the cell's ability to migrate was significantly enhanced (Figure 7(a)) in the scratch test; while after FKBP4 was silenced in A549 cells, the cell's ability to migrate was significantly reduced (Figure 7(b)). These results indicate that FKBP4 can promote the migration of NSCLC cells.

### 3.8. FKBP4 Promotes NSCLC Cell Invasion

**Figure 8:**
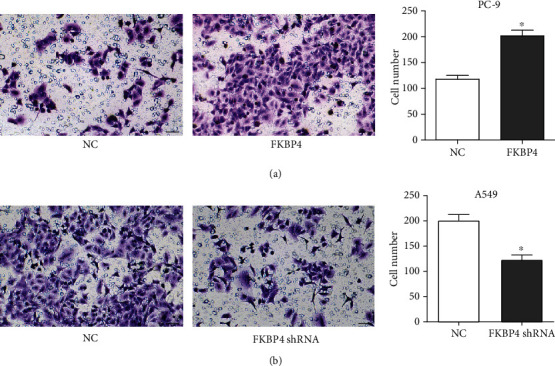
FKBP4 promotes NSCLC cell invasion: cell invasion after overexpression of FKBP4 (a) in PC-9 cells and silence of FKBP4 (b) in A549 cells; *N* = 3, ^∗^*P* < 0.05.

After FKBP4 was overexpressed in PC-9 cells, the invasion ability of the cells was significantly enhanced (Figure 8(a)); whereas after FKBP4 was silenced in A549 cells, the cells' invasion ability was significantly reduced (Figure 8(b)). These results indicate that FKBP4 can promote the invasion of NSCLC cells.

### 3.9. FKBP4 Promotes NSCLC Growth In Vivo

**Figure 9:**
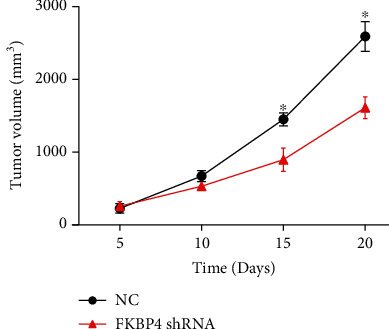
FKBP4 promotes NSCLC growth in vivo; *N* = 3, ^∗^*P* < 0.05.

The results of subcutaneous tumor formation experiments in nude mice demonstrated that after silencing the FKBP4 gene, the tumor growth rate was significantly lower than that of the control group (Figure 9), indicating that tumor growth was inhibited. This result shows that FKBP4 promotes NSCLC growth in vivo.

### 3.10. FKBP4 Activates the Akt/mTOR Signaling Pathway in NSCLC Cells

**Figure 10:**
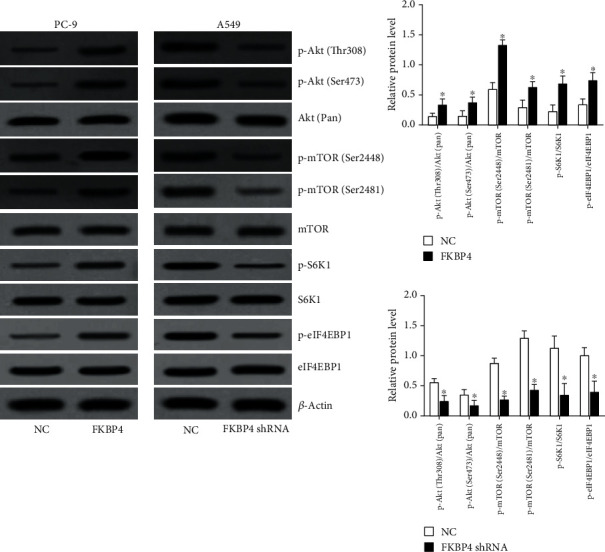
FKBP4 activates the Akt/mTOR signaling pathway in NSCLC cells; *N* = 3, ^∗^*P* < 0.05.

After FKBP4 was overexpressed in PC-9 cells, it is demonstrated that Akt and mTOR phosphorylation levels were significantly increased, while after FKBP4 was silenced in A549 cells, Akt and mTOR phosphorylation levels were significantly reduced (Figure 10). Moreover, the phosphorylation levels of mTOR downstream proteins including S6K1 and eIF4EBP1 were significantly raised by FKBP4 overexpression and reduced by FKBP4 silencing (Figure 10). These results indicate that FKBP4 activates the Akt/mTOR signaling pathway in NSCLC cells.

### 3.11. mTOR Inhibitor Neutralizes the Functions of FKBP4 in NSCLC Cells

**Figure 11:**
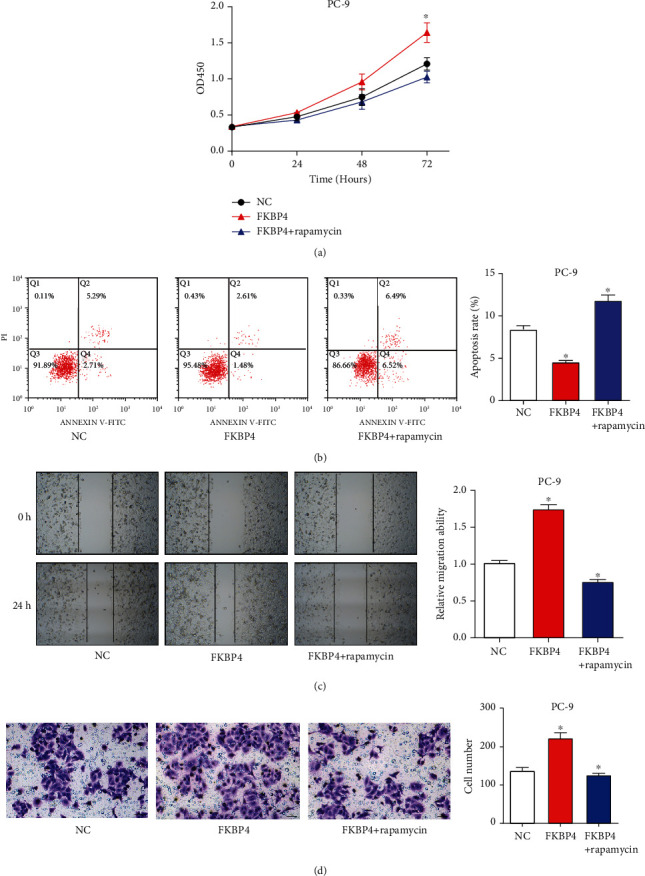
mTOR inhibitor neutralizes the functions of FKBP4 in NSCLC cells. The PC-9 cells transfected with FKBP4 overexpression plasmids were treated with mTOR inhibitor rapamycin (200 nmol/l). Cell proliferation, apoptosis, migration, and invasion were analyzed by the CCK8 assay (a), flow cytometry (b), scratch test (c), and Transwell experiment (d), respectively; *N* = 3, ^∗^*P* < 0.05.

The PC-9 cells transfected with FKBP4 overexpression plasmids were treated with mTOR inhibitor rapamycin (200 nmol/l). It was observed that mTOR inhibitor neutralizes the effects of FKBP4 in promoting proliferation (Figure 11(a)), inhibiting apoptosis (Figure 11(b)), enhancing migration (Figure 11(c)), and invasion of PC-9 cells (Figure 11(d)). These results indicate that the mTOR inhibitor neutralized the functions of FKBP4 in NSCLC cells.

## 4. Discussion

With the continuous improvement of tumor-related databases and the development of bioinformatics analysis technology, more and more oncogenes and tumor suppressor genes are abnormally expressed in a variety of malignant tumors and have been confirmed as biological markers and potential therapeutic targets for tumors [15, 16]. In this study, through bioinformatics analysis, it is found that FKBP4 expression was significantly upregulated in NSCLC and verified by qRT-PCR in NSCLC tissues and cell lines. So far, the expression level of FKBP4 in NSCLC has not been reported, and its clinical significance is not clear. In this study, it is also found that the expression of FKBP4 is closely related to the tumor size and lymph node metastasis of NSCLC, and its high expression suggests a poor prognosis for patients. Therefore, we believe that FKBP4 can be used as a biomarker of NSCLC to indicate the malignant progression of tumors and prognosis and has certain clinical significance.

Since the biological function of FKBP4 in NSCLC is not clear, the effect of FKBP4 on the malignant progression of NSCLC was further explored in NSCLC cells and animal models. The results showed that FKBP4 can promote the proliferation, migration, and invasion of NSCLC cells and inhibit the apoptosis of NSCLC cells in vitro. Also, FKBP4 can promote the growth of NSCLC tumors in vivo. Therefore, we believe that FKBP4 is an oncogene in NSCLC, can promote the malignant process of NSCLC, and may become a therapeutic target for NSCLC.

It is reported that FKBP4 is involved in the pathophysiological process of various diseases. For instance, FKBP4 can increase the current and calcium flux mediated by TRPC3 and activate the calcineurin and T-cell nuclear factor in the transient receptor potential, thereby causing pathological hypertrophy of cardiomyocytes in chronic heart disease [17]. FKBP4 may also be involved in the progesterone resistance process of endometriosis [18]. Patients with Alzheimer's disease have abnormally reduced levels of FKBP4 in the brain and can cause the formation of nerve fiber tangles [19]. In tumors, it has been demonstrated that FKBP4 plays a role in triple-negative breast cancer and castration-resistant prostate cancer [13, 14]; however, the role of FKBP4 in other tumors has not been reported. Therefore, the role of FKBP4 in tumors is largely unknown, and it has a very innovative significance for the research around FKBP4.

The mechanism by which FKBP4 promotes the malignant process of NSCLC was further explored in this study. Our research illustrated that FKBP4 can activate the Akt/mTOR signaling pathway. Previous studies have found that FKBP4 can interact with PI3K in breast cancer and activate Akt through PDK1 and mTORC2, thereby activating the Akt/mTOR signaling pathway [13]. Akt is a protooncogene that regulates various cell functions in tumors, including metabolism, proliferation, survival, migration, and invasion [20]. The activation of Akt depends on the phosphorylation of threonine (Thr) 308 and serine (Ser) 473 [21]. mTOR is an atypical serine/threonine kinase which can promote tumor development by regulating cell biological processes such as metabolism, autophagy, and cellular stress [22]. mTOR involves 2 structurally and functionally distinct signaling complexes mTORC1 and mTORC2. mTORC1 is a growth regulator, which can enhance anabolism or repress catabolism by phosphorylation of substrates, thus promoting cell growth. mTORC2 can phosphorylate the Ser473 site of Akt and activate Akt signaling, thus promoting cell survival [23]. Activated mTORC1 upregulates protein synthesis by phosphorylating key regulators including S6K1 and eIF4EBP1. Our data showed that FKBP4 could promote the phosphorylation of S6K1 and eIF4EBP1 which indicated that FKBP4 might mainly regulate mTORC1 signaling. In summary, we believe that FKBP4 may regulate the biological functions of proliferation, apoptosis, migration, and invasion by activating the Akt/mTOR signaling pathway in NSCLC.

## 5. Conclusion

The results of this study indicate that the expression of FKBP4 is significantly increased in NSCLC and is closely related to the malignant progression of tumors and poor prognosis. FKBP4 can promote the proliferation, migration, and invasion of NSCLC cells by activating the Akt/mTOR signaling pathway, inhibiting NSCLC cell apoptosis, and promoting the growth of NSCLC tumors in vivo. This study suggests that FKBP4 has potential as a biomarker and therapeutic target for clinical application in NSCLC.

## Figures and Tables

**Table 1:** Correlation between clinicopathological factors and FKBP4 expression in patients with NSCLC.

Factors	Number of patients	Upregulated FKBP4	Downregulated FKBP4	*P* value
Gender				
Male	26	12	14	
Female	14	8	6	0.507
Age				
<65	23	12	11	
≥65	17	8	9	0.749
Tumor size				
<3 cm	21	6	15	
≥3 cm	19	14	5	0.004
Lymph node metastasis				
N0	25	9	16	
N1-3	15	11	4	0.022
Grading				
Low	24	11	13	
Medium/advanced	16	9	7	0.519
TNM staging				
I-II	27	11	16	
III-IV	13	9	4	0.091
Smoking history				
No	11	6	5	
Yes	29	14	15	0.723

The chi-square test was used for statistical analysis.

## Data Availability

The data used to support the findings of this study are included within the article.

## References

[1] Siegel R. L., Miller K. D., Jemal A. (2017). Cancer statistics, 2017. *CA: a Cancer Journal for Clinicians*.

[2] Chen W., Zheng R., Baade P. D. (2016). Cancer statistics in China, 2015. *CA: a Cancer Journal for Clinicians*.

[3] Meador Catherine B., Hata Aaron N. (2020). Acquired resistance to targeted therapies in NSCLC: updates and evolving insights. *Pharmacology & Therapeutics*.

[4] Song S., Tan Y. (2019). Expression of FKBP52 in the ovaries of PCOS rats. *International Journal of Molecular Medicine*.

[5] Solassol J., Mange A., Maudelonde T. (2011). FKBP family proteins as promising new biomarkers for cancer. *Current opinion in pharmacology*.

[6] Peattie D. A., Harding M. W., Fleming M. A. (1992). Expression and characterization of human FKBP52, an immunophilin that associates with the 90-kDa heat shock protein and is a component of steroid receptor complexes. *Proceedings of the National Academy of Sciences of the United States of America*.

[7] Ward B. K., Mark P. J., Ingram D. M., Minchin R. F., Ratajczak T. (1999). Expression of the estrogen receptor-associated immunophilins, cyclophilin 40 and FKBP52, in breast cancer. *Breast Cancer Research and Treatment*.

[8] Kumar P., Mark P. J., Ward B. K., Minchin R. F., Ratajczak T. (2001). Estradiol-regulated expression of the immunophilins cyclophilin 40 and FKBP52 in MCF-7 breast cancer cells. *Biochemical and Biophysical Research Communications*.

[9] Periyasamy S., Warrier M., Tillekeratne M. P. M., Shou W., Sanchez E. R. (2007). The immunophilin ligands cyclosporin A and FK506 suppress prostate cancer cell growth by androgen receptor-dependent and -independent mechanisms. *Endocrinology*.

[10] Desmetz C., Bascoul-Mollevi C., Rochaix P. (2009). Identification of a new panel of serum autoantibodies associated with the presence of in situ carcinoma of the breast in younger women. *Clinical Cancer Research*.

[11] Lin J. F., Xu J., Tian H. Y. (2007). Identification of candidate prostate cancer biomarkers in prostate needle biopsy specimens using proteomic analysis. *International Journal of Cancer*.

[12] Liu Y., Li C., Xing Z. (2010). Proteomic mining in the dysplastic liver of WHV/c-myc mice--insights and indicators for early hepatocarcinogenesis. *The FEBS Journal*.

[13] Mangé A., Coyaud E., Desmetz C. (2019). FKBP4 connects mTORC2 and PI3K to activate the PDK1/Akt-dependent cell proliferation signaling in breast cancer. *Theranostics*.

[14] Federer-Gsponer J. R., Quintavalle C., Müller D. C. (2018). Delineation of human prostate cancer evolution identifies chromothripsis as a polyclonal event and FKBP4 as a potential driver of castration resistance. *The Journal of Pathology*.

[15] Liu J., Wan Y., Li S. (2020). Identification of aberrantly methylated differentially expressed genes and associated pathways in endometrial cancer using integrated bioinformatic analysis. *Cancer Medicine*.

[16] Zhang Z., Huang J., Wang G. (2020). Serum miRNAs, a potential prognosis marker of loco-regionally advanced nasopharyngeal carcinoma patients treated with CCRT. *BMC Cancer*.

[17] Bandleon S., Strunz P. P., Pickel S. (2019). FKBP52 regulates TRPC3-dependent Ca signals and the hypertrophic growth of cardiomyocyte cultures. *Journal of Cell Science*.

[18] Joshi N. R., Miyadahira E. H., Afshar Y. (2017). Progesterone resistance in endometriosis is modulated by the altered expression of microRNA-29c and FKBP4. *The Journal of Clinical Endocrinology and Metabolism*.

[19] Meduri G., Guillemeau K., Dounane O. (2016). Caspase-cleaved tau-D(421) is colocalized with the immunophilin FKBP52 in the autophagy-endolysosomal system of Alzheimer’s disease neurons. *Neurobiology of Aging*.

[20] Mirza-Aghazadeh-Attari M., Ekrami E. M., Aghdas S. A. M. (2020). Targeting PI3K/Akt/mTOR signaling pathway by polyphenols: implication for cancer therapy. *Life Sciences*.

[21] Yudushkin I. (2020). Control of Akt activity and substrate phosphorylation in cells. *IUBMB Life*.

[22] Zou Z., Tao T., Li H., Zhu X. (2020). mTOR signaling pathway and mTOR inhibitors in cancer: progress and challenges. *Cell & Bioscience*.

[23] Ferrín G., Guerrero M., Amado V., Rodríguez-Perálvarez M., de la Mata M. (2020). Activation of mTOR signaling pathway in hepatocellular carcinoma. *International Journal of Molecular Sciences*.

